# The Utility of the Knotless Suture Fixation for Bilateral Second Toe Transplantation in Traumatic Multiple-Digit Amputation

**DOI:** 10.1155/2018/5194918

**Published:** 2018-10-18

**Authors:** Yasuhiro Yamamoto, Satoshi Ichihara, Akira Hara, Toshiya Kudo, Yuichiro Maruyama, Hajime Kajihara, Kazuo Kaneko

**Affiliations:** ^1^Department of Orthopedic Surgery, Koto Hospital, Tokyo, Japan; ^2^Hand Surgery Center, Juntendo University Urayasu Hospital, Chiba, Japan; ^3^Department of Orthopedic Surgery, Juntendo University Urayasu Hospital, Chiba, Japan; ^4^Department of Orthopedic Surgery, Juntendo University, Tokyo, Japan

## Abstract

Toe-to-hand transfer is a useful reconstruction method after finger amputation. We report a case of multiple-digit amputation, reconstructed with bilateral second-toe transfer. In this study, we used a knotless suture fixation system (ZipTight™; Arthrex Inc., FL, USA) which effectively closed the wound and reduce the amount of dead space. Both second-toe transplantations survived. The feet were asymptomatic with good cosmetic outcomes. Although the reconstructed digits had limited range of motion, the patient was able to return to work. Knotless suture fixation system may be one of the effective methods for closing the donor site wound in second-toe transplantations.

## 1. Introduction

At the end of the 19th century, Nicoladoni [1, 2] described the first case of pedicle toe-to-hand transfer for thumb reconstruction. Toe-to-finger reconstruction was also described by Blair and Byars [3] in 1940. Cobbet [4] performed the first free transfer of great toe-to-thumb in 1968. With improvements in instruments and microscopes, microsurgery has rapidly expanded and progressed in several fields [5].

In patients with injury to multiple digits, reconstruction of hand functions such as the tripod pinch, strong hook grip, and precise hand motion is important [6, 7]. In recent microsurgical reconstructions of multiple-digit injury, instead of single-toe transplantation for thumb or finger reconstruction, multiple digits have been reconstructed with the use of bilateral toe transplantation [8], combined second- and third-toe transplantation [9], and transplantation of different toe combinations [10]. We herein describe the feasibility of bilateral second-toe transplantation (STT) in a patient with multiple-digit amputation.

## 2. Case Presentation

**Figure 1:**
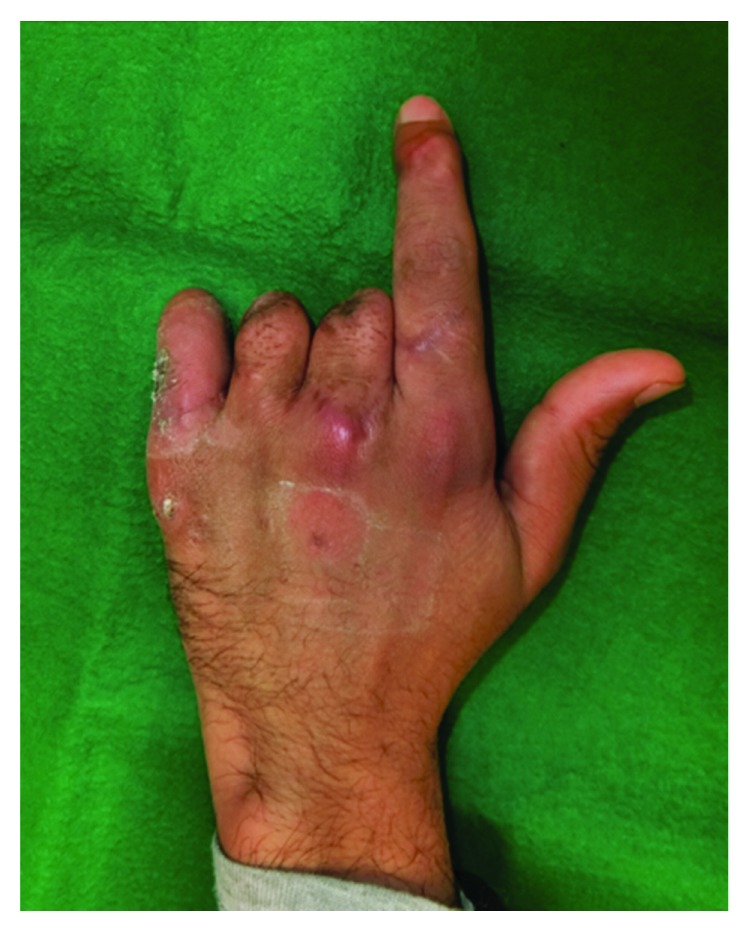
The Left hand after amputation. The middle and ring fingers were amputated proximal to the proximal interphangeal joint and, the small finger was amputated distal to the proximal interphalangeal joint.

**Figure 2:**
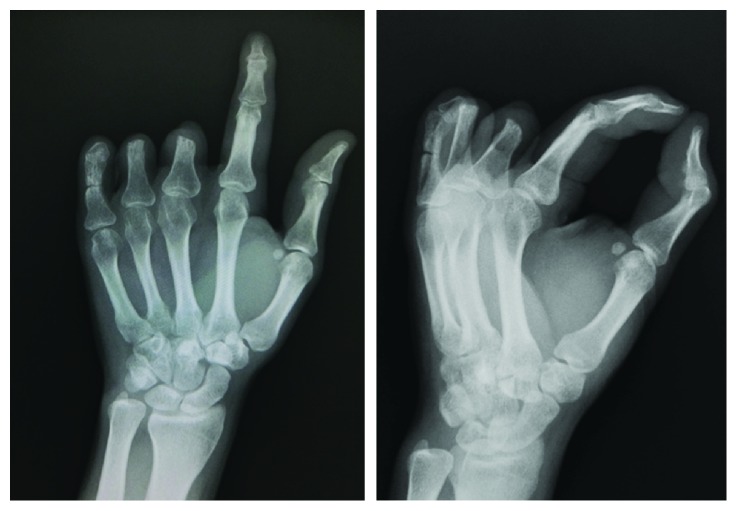
Preoperative X-ray confirms findings based on hand appearance. Nonunion of the proximal phalanx is seen.

**Figure 3:**
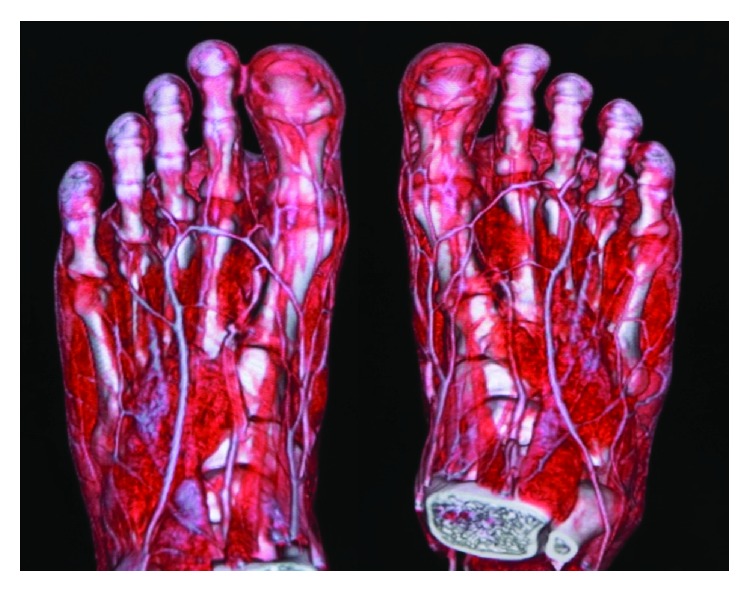
Enhanced computed tomography was used to evaluate toe vasculature before surgery.

The left hand of a right-handed 29-year-old man was injured by a meat chopper. The injured fingers were not replantable; therefore, amputation of the middle and ring fingers at the level of the proximal phalanx and of the little finger at the middle phalanx was performed at another hospital (Figure 1). The patient's occupation was chef at an Indian restaurant. Six months following the injury, the patient was referred to our hospital for hand reconstruction. Radiographic images confirmed the clinical findings (Figure 2). The patient's preoperative visual analogue scale (VAS) score was 4/10 (this score is based on the patient's phantom pain after the finger amputations) and his Quick-DASH was 81.82/100.00. Examination of foot vascularity with contrast-enhanced computed tomography confirmed that bilateral STT was compatible for the reconstruction of two fingers (Figure 3). Therefore, one year after the injury, the bilateral second toes were transferred to the middle and ring fingers.

### 2.1. Finger Dissection

**Figure 4:**
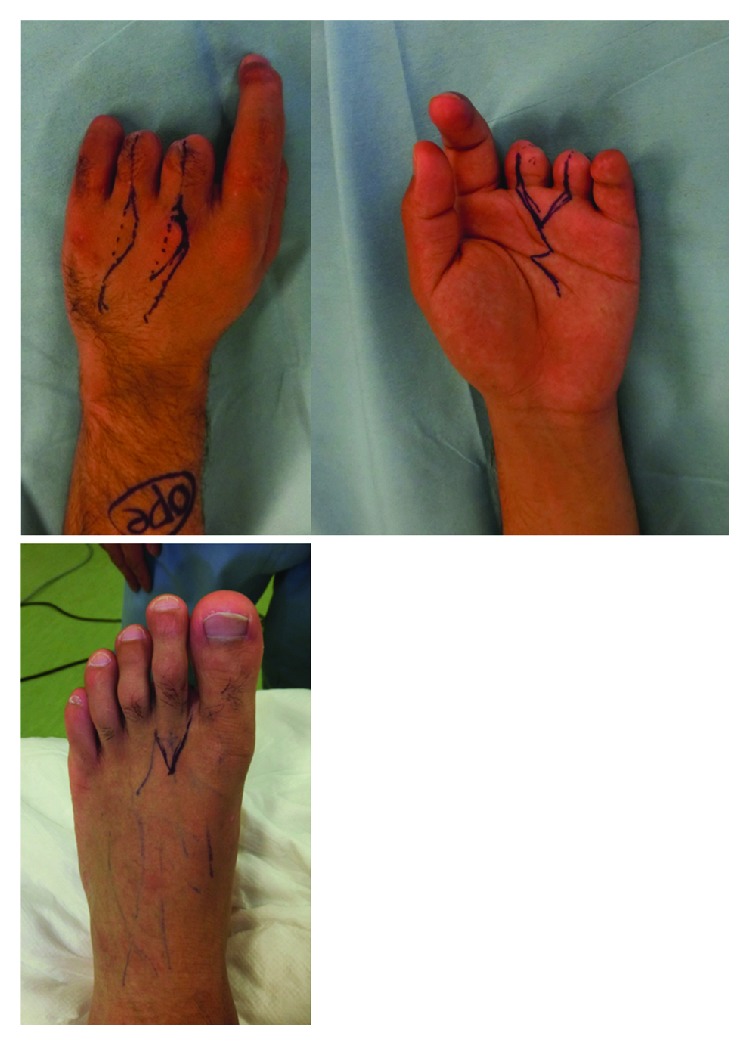
Design of the hand. We confirmed the dorsal metacarpal vein with ultrasonography.

A curved incision was made over the volar surface of the distal middle and ring fingers (Figure 4). We identified the digital artery, digital nerve, and flexor digitorum profundus on the volar side. The digital artery and digital nerve were isolated to provide for inflow and reinnervation.

### 2.2. Foot Dissection

**Figure 5:**
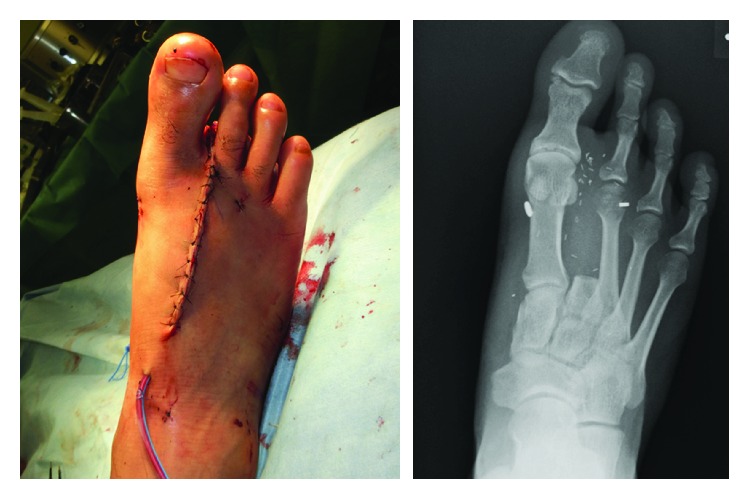
Closure of right donor site with a knotless suture fixation system (ZipTight™). The device tightened the space between the metatarsal bones of the first and third toes.

First, the dorsalis pedis artery and superficial dorsal vein were marked under ultrasound guidance. A v-shaped incision was made at the base of the second toe and extended proximally. The superficial dorsal veins, first dorsal metacarpal artery, and extensor digitorum longus were dissected on the dorsal side. A plantar dissection was also made, and the flexor digitorum longus and proper palmer digital nerves were identified. Disarticulation was performed at the second metatarsophalangeal joint. While harvesting the second toe from the recipient artery, long pedicle was maintained in order to facilitate vascular anastomosis and to avoid the kinking of the artery. The second metatarsal bone was cut at the base to adjust the length of fingers. The foot was closed with a knotless suture fixation system (ZipTight™; Arthrex Inc., FL, USA) to firmly close the wound, avoiding unwanted reduction of tension during the closure (Figure 5).

## 3. Toe Transplantation

**Figure 6:**
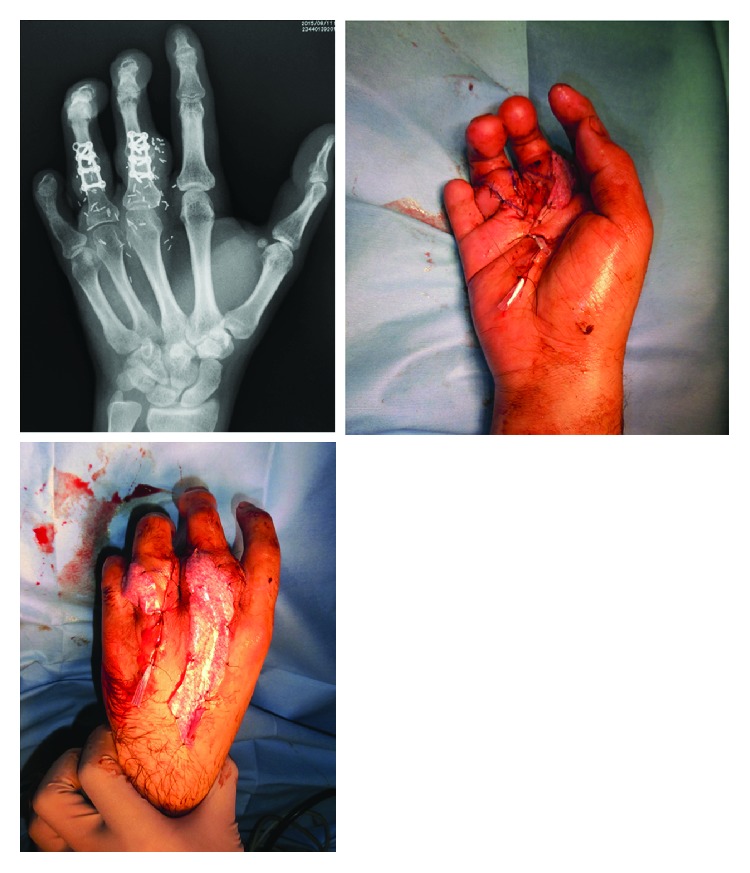
X-ray of the hand after transplantation. The second toe and proximal phalanx are fixed with a plate.

The bilateral toes were fixed in the anatomic position to the proximal phalanx of the middle and ring fingers with a nonlocking plate (Modular Hand System; Synthes Inc., Zuchwil, Switzerland). The flexor and extensor tendons of both fingers were approximated to the corresponding flexor digitorum longus and extensor digitorum longus with interlacing suture. The bilateral plantar nerves were anastomosed under the microscope to the volar digital nerves of the middle and ring fingers. The veins and arteries at the donor site were anastomosed to the dorsal veins and digital arteries with 10-0 suture (Figure 6). After flow was confirmed, the area was closed with local skin flaps and a split-thickness skin graft from the volar forearm. A volar splint was applied for 2 weeks to prevent ischemia and necrosis of the skin graft.

## 4. Results

**Figure 7:**
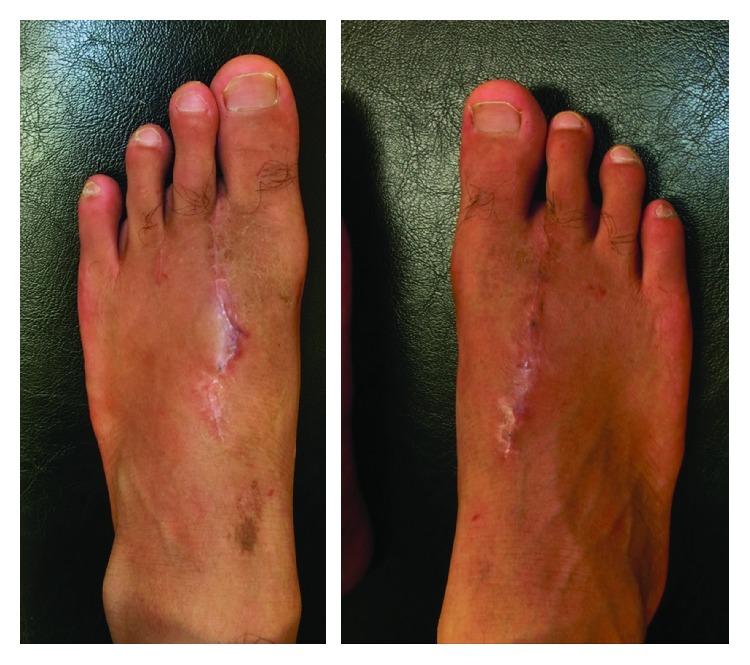
Donor site deformity that goes unnoticed. There were no complications involving dehiscence, pain, or gait disturbance.

**Figure 8:**
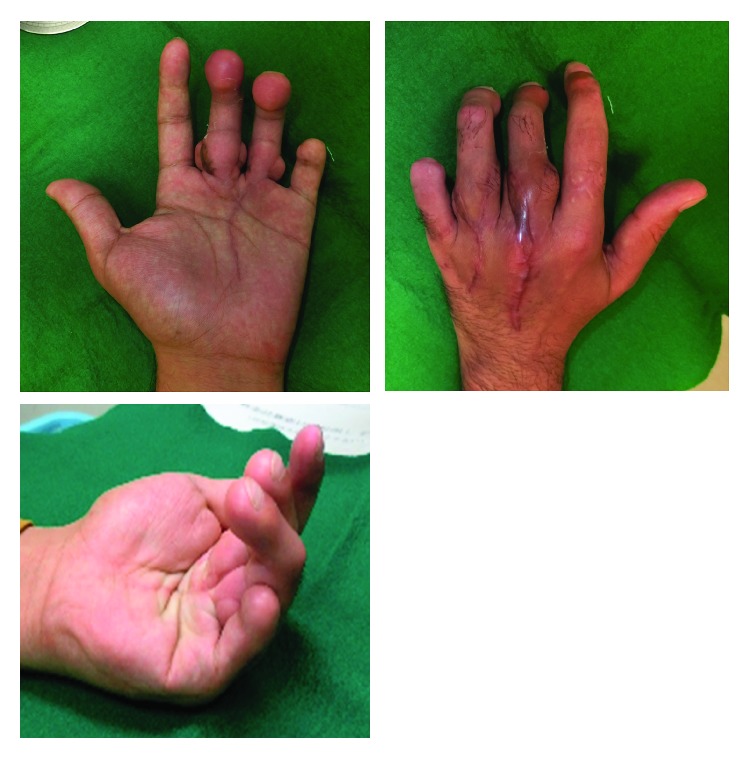
Postoperative extension and flexion appearance of the left hand. Good results are seen at 8-month follow-up after bilateral second toe transplantation. The patient is satisfied with the results.

Both STTs survived and bone union was achieved. Eight months after the surgery, the total active motion of the middle finger was 65 degrees (total passive range of motion was 70 degrees) and that of the ring finger was 70 degrees (total passive range of motion was 80 degrees). Both fingers had sensitivity to the blue filament on the Semmes–Weinstein test. Approximately 12 months after the transplantation, the patient's VAS score was 1/10 and his Quick-DASH score was 18.18/100.0. He regained appropriate function of his fingers using the reconstructed fingers. No foot disability or gait disturbances occurred. The left foot was asymptomatic and had a good cosmetic outcome (Figure 7). Although the patient had limited range of motion in the fingers, he returned to work as a chef and was satisfied with the cosmetic results (Figure 8).

## 5. Discussion

Patients who undergo digit amputation not only suffer from hand disability but also from psychological damage due to cosmetic concerns. Multiple-digit amputation can cause especially serious problems. Therefore, the anatomical reconstruction of injured digits is mandatory [11]. Toe-to-hand transfer is a standard procedure for traumatic thumb injury, digit amputation distal to the flexor digitorum superficialis insertion, and traumatic multiple-digit amputation [7]. When amputation of multiple digits occurs at a level proximal to the flexor digitorum superficialis insertion, patients generally lose prehensile ability [12]. The feet and hands are anatomically similar in form and structure and feet are suitable for use in hand reconstruction. The advantages of hand reconstruction with the use of feet include (1) recovery of functions such as the tripod pinch, hook grip, and grasp, (2) cosmetic repair, and (3) early return to work. It is important to select appropriate reconstruction methods, depending on the number and location of amputations. The thumb, index finger, and middle finger are important to achieve adequate pinching function. The middle and ring fingers are important to achieve a powerful grip. In cases of multiple-digit amputation at a level distal to the web, bilateral STT is preferred because unilateral transfer of the second and third toes combined would create the appearance of syndactyly. However, in the case of multiple-digit amputation at a level proximal to web, unilateral transfer of the second and third toes combined is preferred [6, 13]. As the amputation previously performed on the patient was distal to the web, on the basis of previous reports, we performed bilateral STT to the middle and ring fingers in order to recover appropriate grip strength. Problems associated with skin coverage and wound healing are among the most serious complications in toe-to-finger transplantation. Sosin et al. [14] reported differences in functional impairment of the foot after operation and the hand reoperation rate between STT, great-toe transfers (GTT), and combined-toe transfers (CTT). STT scored the lowest in the functional impairment (STT, 14.5%; GTT, 21.8%; and CTT, 23.0%); however, it scored the highest in the hand reoperation rate (STT, 16.6%; GTT, 4.5%; and CTT, 16.0%). The authors reported that complex procedures in STT and CTT resulted in functional impairments at donor site closure. Rajendra et al. [15] described reducing the gap between the first and third toes after harvesting of the second toe, with repair of the intermetatarsal ligament and transverse K-wire to avoid dehiscence. We employed a similar procedure however, repairing the intermetatarsal ligament using a knotless suture fixation system (ZipTight™). This less invasive procedure might be more appreciated and useful in preventing postoperative complications such as wound dehiscence and toe deformity. Maruccia et al. [16] reported migration of the fourth toe toward the scissoring deformity 31 years after combined second and third toe transfer. The patient complained of discomfort and pain while walking. CTT may be more destructive than STT over time. We think that STT causes less donor site morbidity than CTT. When reconstructing multiple-digit amputations, we recommend bilateral STT.

## 6. Conclusion

We performed bilateral STT on a patient with multiple-digit amputation. Knotless suture fixation system is one of effective methods for closing the donor site wound in second-toe transplantations.

There were no serious complications in either foot, and hand function was restored, allowing the patient to return to work.

## References

[1] Nicoladoni C. (1897). Daumenplastik. *Wiener Klinische Wochenschrift*.

[2] Nicoladoni C. (1900). Daumenplastik und organischer Ersatz der Fingerspitze (Anticheiroplastik und Daktyloplastik). *Arch fur Klinische Chirugie*.

[3] Blair V. P., Byars L. T. (1940). Brief communications and case reports. Toe to finger transplant. *Annals of Surgery*.

[4] Cobbett J. R. (1969). Free digital transfer: report of a case of transfer of a great toe to replace an amputated thumb. *Journal of Bone and Joint Surgery*.

[5] Li X., Cui J., Maharjan S., Yu X., Lu L., Gong X. (2016). Neo-digit functional reconstruction of mutilating hand injury using transplantation of multiple composite tissue flaps. *Medicine*.

[6] Wei F.-C., el-Gammal T. A., Lin C.-H., Chuang C.-C., Chen H.-C., Chen S. H. T. (1997). Metacarpal hand: classification and guidelines for microsurgical reconstruction with toe transfers. *Plastic and Reconstructive Surgery*.

[7] Waljee J. F., Chung K. C. (2013). Toe-to-hand transfer: evolving indications and relevant outcomes. *The Journal of Hand Surgery*.

[8] O'brien B. M., Brennen M. D., Macleod A. M. (1978). Simultaneous double toe transfer for severely disabled hands. *The Hand*.

[9] Vergara-Amador E. (2015). Second toe-to-hand transplantation: a surgical option for hand amputations. *Colombia Médica*.

[10] Chen H. C., Tang Y. B., Wei F. C., Noordhoff M. S. (1991). Finger reconstruction with triple toe transfer from the same foot for a patient with a special job and previous foot trauma. *Annals of Plastic Surgery*.

[11] Brooks D., Buntic R. F., de Jesus R. (2008). Creation of a four-joint-digit after second toe to digit transplantation: restoration of form and function. *Microsurgery*.

[12] Lin C. H., Hu T. L., Lin C. H. (2008). Split second- and third-toe transplantation in mutilating-hand-injury reconstruction. *Annals of Plastic Surgery*.

[13] Tsai T.-M., Jupiter J. B., Wolff T. W., Atasoy E. (1981). Reconstruction of severe transmetacarpal mutilating hand injuries by combined second and third toe transfer. *The Journal of Hand Surgery*.

[14] Sosin M., Lin C.-H., Steinberg J. (2016). Functional donor site morbidity after vascularized toe transfer procedures: a review of the literature and biomechanical consideration for surgical site selection. *Annals of Plastic Surgery*.

[15] Nehete R., Nehete A., Singla S., Adhav H. (2012). Bilateral microvascular second toe transfer for bilateral post-traumatic thumb amputation. *Journal of Plastic Surgery*.

[16] Maruccia M., Kiranantawat K., Yeo M. S. W., Nicoli F., Ciudad P., Chen H. C. (2014). Donor site of toe transfer: is combined second and third toe transfer the better choice? A 31 years of long-term follow up. *Microsurgery*.

